# Application of a Bioactive Compound 2,4-Di-tert-butylphenol in Nanoemulsion Form for Shelf-Life Extension of Cherry Tomatoes: From Microbial Inactivation to Quality and Safety Evaluation

**DOI:** 10.3390/foods15172966

**Published:** 2026-08-24

**Authors:** Yanxin Zhang, Hui Li, Chenxi Yan, Liran Yang, Meng Zhou, Zhongyao Chen, Yukou Li, Shuang Jia, Dongming Li, Jianchun Qin

**Affiliations:** 1 Key Laboratory of Herbage and Endemic Crop Biology, School of Life Sciences, Ministry of Education, Inner Mongolia University, Hohhot 010070, China; zhangyanxin@imu.edu.cn (Y.Z.); lihui200206@163.com (H.L.); 19322109129@163.com (C.Y.); 15253182860@163.com (L.Y.); mengzhou1224@163.com (M.Z.); 17738005615@163.com (Z.C.); 19355354496@163.com (Y.L.); s_jia@imu.edu.cn (S.J.); 2 College of Plant Sciences, Jilin University, Changchun 130062, China

**Keywords:** bioactive compound, 2,4-di-tert-butylphenol, nanoemulsion, postharvest preservation, antimicrobial

## Abstract

Postharvest spoilage of cherry tomatoes caused by pathogenic microorganisms and oxidative browning leads to significant economic losses and food waste. Natural bioactive compounds are gaining increasing attention as alternatives to synthetic pesticides. In this study, a nanoemulsion (NED) formulation of nature-derived 2,4-di-tert-butylphenol was developed and evaluated for its potential to preserve cherry tomato quality during storage. The NED was prepared using high-pressure homogenization, and demonstrated good water solubility and stability. The particles and polydispersity index of NED were an average size of 80–170 nm and 0.2389, respectively. The conductivity and zeta potential of the nanoemulsion were detected as 1.007 mS/cm and 1.611 mV, respectively. Antimicrobial assays showed that the nanoemulsion effectively inhibited the growth of major postharvest pathogens, including human-pathogenic bacterial *S. aureus*, and phytopathogenic fungal *B. cinerea*, with minimum inhibitory concentration values of 1.0, 2.0 μL/mL, respectively. Preservation performance tests indicated that the nanoemulsion was effective under both room temperature and refrigerated conditions. When applied to cherry tomatoes, the NED significantly reduced surface bacterial quantity, and preserved fruit quality, as evidenced by a lower weight loss rate, and higher levels of soluble sugars, protein, total phenolics, flavonoids, and ascorbic acid, and total antioxidant capacity (all comparisons were statistically evaluated by one-way ANOVA followed by Tukey’s HSD test, *p* < 0.05). Importantly, the NED can be completely removed after three times of washing. Collectively, these findings demonstrate that NED is an effective and safe bioactive preservative under laboratory-scale conditions. However, further validation under commercial postharvest handling conditions is necessary prior to practical application. This work provides a fundamental basis for the application of nanoencapsulated natural phenolic compounds in the postharvest preservation of fruits and vegetables.

## 1. Introduction

Postharvest spoilage of fresh horticultural crops represents a major constraint on global food security, economic sustainability, and public health [1,2]. According to the FAO, fruits and vegetables suffer the highest postharvest losses among all agricultural commodity groups, reaching 25.4% of total production, and losses of perishable crops such as tomatoes can be as high as 50% in developing countries [3,4,5]. Among the numerous factors contributing to these losses, microbial decay and oxidative deterioration have been identified as the dominant drivers, accounting for the majority of waste throughout the supply chain [6,7]. Microbial spoilage accounts for approximately 25% of food loss and waste at the global level [8]. Previous studies have reported that the spoilage-associated microorganisms include bacterial pathogens such as *Staphylococcus aureus* and *Escherichia coli* [9,10], as well as fungal pathogens like *Botrytis cinerea*, *Colletotrichum acutatum*, *Rhizopus stolonifer*, *Alternaria alternata* and *Penicillium expansum* [11,12,13,14,15]. *E. coli* and *S. aureus* contamination on the surfaces of fruits and vegetables constitutes a significant risk factor for foodborne diseases, which can lead to severe consequences such as hemorrhagic diarrhea [16,17]. Such contamination is frequently linked to inadequate implementation of Good Agricultural Practices (GAPs) during primary production, including the use of contaminated irrigation water, poor field sanitation, and insufficient hygiene management during harvesting and handling. Even on GAP-certified farms, studies have detected *S. aureus* and *E. coli* in soil and agricultural water [18], indicating that current GAPs do not always eliminate microbial risks. These persistent contamination issues underscore the need for additional interventions beyond GAP implementation to ensure the microbial safety of fresh produce. Moreover, fungal contamination also poses a significant threat to human health; for instance, several fungi, including *Botrytis*, *Alternaria*, *Penicillium*, and *Aspergillus*, can produce a range of mycotoxins, such as patulin, ochratoxin A, and alternariol, all of which have been demonstrated to be cytotoxic, genotoxic, and potentially carcinogenic [19,20]. Given these multifaceted risks, research into storage preservation technologies is of great importance for maintaining fruit and vegetable quality and ensuring food safety. Focusing specifically on cherry tomatoes, they are particularly susceptible to microbial infection and oxidative injury owing to their thin pericarp, high water and nutrient content, and rapid ripening after harvest [21,22]. It has been reported that fruit decay caused by microbial pathogens accounts for 20–30% of postharvest losses worldwide, and in some developing regions this figure can exceed 40%, severely compromising cherry tomato quality and marketability [23,24]. These realities underscore the urgent need for effective, safe, and sustainable strategies to mitigate postharvest decay and extend the shelf life of cherry tomatoes.

Low-temperature storage is widely recognized as the primary strategy for extending the postharvest life of fresh fruits and vegetables. However, the efficacy of cold storage is highly dependent on the availability and continuity of the cold-chain. Studies have demonstrated substantial differences in quality retention between refrigerated and ambient storage. For instance, after 12 days of storage, weight loss in green chili was limited to 4.7% under refrigerated conditions, compared with 25.2% under ambient conditions [25]. At the global level, the FAO estimates that 526 million tonnes of food are lost annually due to inadequate refrigeration and storage conditions. In developing regions, where cold chain coverage remains limited, postharvest losses of fruits and vegetables can reach 40–50% of production, rising above 50% for highly perishable crops [26]. These figures highlight that while cold storage is effective, its benefits are not universally accessible, underscoring the urgent need for complementary preservation strategies that can function independently of continuous refrigeration.

In response to growing concerns over microbial resistance and chemical residues associated with synthetic pesticides, natural phenolic compounds have emerged as promising alternatives in the agricultural and food industries [27,28]. These compounds not only inhibit microbial growth but also possess antioxidant properties, thereby extending the shelf life and safety of food products, making them highly attractive candidates for postharvest preservation [29]. The antimicrobial and antioxidant activities of phenolic compounds are largely attributed to their ability to disrupt microbial cell membranes, interfere with essential enzymatic processes, and scavenge free radicals [30,31]. To date, numerous small-molecule phenolics have been successfully applied in fruit preservation [32]. For example, caffeic acid, ferulic acid, and gallic acid have been shown to suppress mycelial growth and spore germination of postharvest fungal pathogens such as *Colletotrichum* and *Penicillium* [33,34]. Likewise, thymol and carvacrol, which are major constituents of oregano and thyme essential oils, exhibit strong antibacterial activity against foodborne pathogens including *S. aureus* and *E. coli*, and have been incorporated into edible coatings for fresh produce [35,36,37].

Among these naturally occurring phenolic compounds, 2,4-di-tert-butylphenol (2,4-DTBP) is a hindered phenolic substance that has been isolated from various plant sources and microbial metabolites. Importantly, it displays both potent antibacterial and antioxidant activities, and has demonstrated fungicidal effects against *Aspergillus niger*, *Fusarium oxysporum*, and *Penicillium chrysogenum* [38,39]. Moreover, wheat grains treated with 2,4-DTBP showed increased resistance to fungal attack, further supporting its potential as a natural preservative [40]. Despite these favorable properties, the practical application of 2,4-DTBP, similar to many hydrophobic phenolic compounds, is hindered by poor water solubility and limited dispersion stability. To overcome this bottleneck, nanoemulsion technology offers an effective solution. Specifically, nanoemulsions are kinetically stable colloidal delivery systems with droplet sizes typically ranging from 20 to 200 nm. They can encapsulate lipophilic bioactive compounds, improve their aqueous dispersibility, and enhance their bioavailability and sustained-release properties [41].

The efficacy of this approach has been corroborated by recent studies. For instance, Sun and colleagues reported that a composite film incorporating ginger essential oil nanoemulsion extended the shelf life of cherry tomatoes by approximately 6 days during a 15-day storage period, with no fungal growth observed throughout the entire storage duration [42]. In another study, bergamot essential oil nanoemulsion embedded in chitosan/gum arabic coatings more than doubled the shelf life of cherry tomatoes by inhibiting microorganisms, reducing water loss, and mitigating decay [43]. Similarly, a chitosan-based nanoemulsion containing *Phoebe bournei* wood essential oil achieved 100% inhibition of gray mold lesions on cherry tomatoes [44]. Taken together, these findings clearly indicate that nanoemulsion encapsulation represents a highly promising strategy to overcome the water-insolubility limitations of hydrophobic phenolic compounds and to unlock their full potential in postharvest preservation.

Although the antimicrobial and antioxidant activities of 2,4-DTBP have been individually recognized, and nanoemulsion technology has been independently validated as an effective delivery system for other phenolic compounds, the combination of these two approaches, specifically the development and comprehensive evaluation of a 2,4-DTBP nanoemulsion for fruit preservation, remains unexplored. To the best of our knowledge, no systematic study has yet been conducted on the application of 2,4-DTBP nanoemulsion for the postharvest preservation of cherry tomatoes. Consequently, this work represents systematic investigation into the use of 2,4-DTBP nanoemulsion as a novel bioactive preservative for cherry tomatoes. Specifically, we prepared and characterized a stable 2,4-DTBP nanoemulsion using high-pressure homogenization, and evaluated its in vitro antimicrobial activity against major postharvest pathogens. Furthermore, we assessed the preservation performance of the nanoemulsion on cherry tomatoes under both room temperature and refrigerated storage conditions by monitoring surface microbial counts, as well as fruit quality parameters. Finally, we determined the removal pattern of 2,4-DTBP on cherry tomatoes after washing, thereby providing a safety basis for practical application. In summary, this study aims to establish a scientific foundation for the development of 2,4-DTBP nanoemulsion as an effective, safe, and sustainable alternative to synthetic pesticides for postharvest fruit preservation.

## 2. Materials and Methods

### 2.1. Materials

Cherry tomatoes (*Solanum lycopersicum* var. cerasiforme) at the fully ripe stage, uniform in size and free from visible mechanical damage, were purchased from Jiajiayue Supermarket (Hohhot, Inner Mongolia, China) and used for subsequent experiments.

The bacterial strain *S. aureus* (ATCC 6538) was obtained from the Microbiology Laboratory of Inner Mongolia University, while the fungal strain *B. cinerea* (ATCC 12481) was obtained from the Laboratory of Secondary Metabolism and Genetic Development at Jilin University.

### 2.2. Reagents

LB broth, LB agar, potato dextrose agar, bovine serum albumin and Coomassie Brilliant Blue G-250 were purchased from Beijing Coolaber Technology Co., Ltd. (Beijing, China) Tween 80, 2,4-di-tert-butylphenol, 4,7-diphenyl-1,10-phenanthroline, trichloroacetic acid, phenol, ferric chloride, ascorbic acid, gallic acid, 2,2-Diphenyl-1-picrylhydrazyl (DPPH) and sodium carbonate were purchased from Shanghai Micron Biotechnology Co., Ltd. (Shanghai, China). Sulfuric acid, ethyl acetate, peptone, sodium chloride, hydrochloric acid, methanol and sucrose were purchased from Nanjing Chemical Reagents Co., Ltd. (Nanjing, China) Folin–Ciocalteu was purchased from Shanghai Beyotime Biotechnology Co., Ltd. (Shanghai, China).

### 2.3. Preparation and Characterization of 2,4-DTBP Nanoemulsion (NED)

The preparation of NED followed the protocol established by Song et al. [45], employing ethyl acetate as the solvent for 2,4-DTBP and Tween 80 as the emulsifier, with the process relying primarily on high-pressure homogenization technology. The entire fabrication procedure encompassed five successive stages: dissolution, mixing, degassing, pre-emulsification, and homogenization. In detail, a 1 g/mL solution of 2,4-DTBP was first prepared, to which Tween 80 was added at a volume corresponding to 10% of that of the 2,4-DTBP solution, and the mixture was subjected to magnetic stirring at 60 °C for 4 h to ensure thorough incorporation. Subsequently, dissolved gases were removed via ultrasonic treatment. The resulting mixture was then combined with deionized water at a volume ratio of 1:8 under continuous stirring at 45 °C, followed by ultrasonication for 40 min to accomplish pre-emulsification. Finally, the pre-emulsion was passed through a high-pressure homogenizer (Phd D-3L, VELP, Usmate Velate, Italy) at 20,000 psi for 8 cycles to yield the NED. The as-obtained formulation was subjected to full-wavelength scanning to characterize its UV-Vis (UV755B, Shanghai Keyou Instrument & Meter Co., Ltd., Shanghai, China) absorption profile, and its mean particle size, polydispersity index, and zeta potential were determined using a zetasizer advance (Malvern, Worcestershire, UK).

### 2.4. In Vitro Antimicrobial Activity Testing of NED

*S. aureus* and *B. cinerea* were selected as the test indicator strains, the former being a mandatory microbiological testing item for ready-to-eat fruit and vegetable products as stipulated by the Chinese national standard GB 29921-2021, National food safety standard—Limits of pathogenic bacteria in prepackaged foods [46], and the latter representing the predominant postharvest pathogenic fungus responsible for tomato decay [47]. The antimicrobial activities of NED were evaluated using the poison medium method, following the protocol described by a previous report [48]. In brief, varying aliquots of NED were incorporated into potato dextrose agar or LB agar with thorough mixing to prepare poisoned plates at final concentrations of 0.2, 0.4, 0.6, 0.8, 1.0, 1.2, 1.4, 1.6, 1.8, and 2.0 μL/mL, respectively. Blank control plates without NED and positive control plates containing 5 μg/mL carbendazim (a common fungicide used in agriculture) or 1 μg/mL ampicillin (an antibiotic commonly used in medicine for treating *S. aureus* infections) were also prepared. For *S. aureus*, 50 μL of bacterial suspension (10^6^-fold diluted suspension with OD_600_ = 1.2) was evenly spread onto LB agar plates with a diameter of 6 cm, and after incubation at 37 °C for 16 h, the number of colony-forming units was enumerated, from which the inhibition rate was calculated according to Equation (1).

(1) $$\mathrm{Inhibition}\;\mathrm{rate}\;(\%)=\frac{Nc-Nt}{Nc}\times 100\%$$

where *Nc* and *Nt* represent the colony counts of the control and treatment group, respectively.

For *B. cinerea*, mycelial plugs of 0.5 cm in diameter were excised with a cork borer, inoculated onto the center of PDA plates, and incubated at 25 °C for 3 d. The colony diameters were then measured using the cross-streaking method, and the inhibition rates were derived from Equation (2).

(2) $$\mathrm{Inhibition}\;\mathrm{rate}\;(\%)=\frac{Dc-Dt}{Dc-0.5}\times 100\%$$

*Dc* and *Dt* represent the colony diameters (cm) of the control and the test group, respectively.

### 2.5. Preservation Efficacy Test of NED on Cherry Tomatoes

The preservation efficacy of NED was evaluated following the previous research [49]. A total of 20 cherry tomatoes of uniform size and free from visible mechanical damage were selected, gently brushed to remove adhering debris and soil, and weighed to record their initial fresh weight. The fruits were then surface-sterilized by immersion in 75% ethanol for 10 min, after which they were removed and blotted dry with sterile gauze. Subsequently, the tomatoes were inoculated by submersion in a bacterial suspension of *S. aureus* adjusted to 10^8^ CFU/mL (OD_600_ = 1) for 40 min, taken out, and left on sterile gauze until surface moisture had evaporated. The inoculated fruits were then immersed in either sterile distilled water or NED solutions at varying concentrations (1, 2, and 4 μL/mL) for 1 h, drained, transferred into sterile sampling bags, and stored at either 4 °C or 25 °C for 7 d, during which their visual quality was monitored periodically. For recovery of surface-attached microorganisms, 10 mL of 1% peptone solution was added to each bag and used to rinse the fruit surface repeatedly; this rinsing procedure was carried out four times, and all washing fluids were pooled and centrifuged at 5000 rpm for 5 min at 4 °C to harvest the cell pellets, which were then resuspended in 5 mL of LB broth. A total of 50 μL of each suspension (diluted 10^5^-fold for the 25 °C-stored groups, whereas without dilution for the 4 °C-stored groups) was spread onto LB agar plates and incubated at 37 °C for 14 h, after which the number of colony-forming units was counted and converted to bacterial concentration according to Equation (3).

(3) $$\mathrm{Concentration}\;\mathrm{of}\;\mathrm{bacterium}\;(\mathrm{cfu}/\mathrm{mL})=\frac{Np\times 100\times n}{40}$$

*Np* represents the number of colonies on the plate, and n denotes the dilution factor.

After microbial removal, the tomatoes were wiped dry, reweighed, and the weightlessness rate was computed using Equation (4).

(4) $$\mathrm{Weightlessness}\;\mathrm{rate}\;(\%)=\frac{m_{b}-m_{a}}{m_{b}}\times 100\%$$

In the formula, *m_a_* represents the mass of fresh tomatoes before processing, and m_b_ represents their mass after storage.

For the *B. cinerea* inoculation group, the fruits were pre-treated by immersion in sterile water or NED solutions for 1 h, after which a small wound was made at the equatorial region using a sterile needle, and 10 μL of fungal spore suspension with a concentration of 10^6^ CFU was inoculated into each wound. The inoculated fruits were subsequently stored at 4 °C or 25 °C for 7 d with periodic observations of disease progression. At the end of the storage period, the fruits were weighed for calculation of weightlessness rate, and the decay rate was determined according to Equation (5), with fruits being scored as decayed when the lesion diameter exceeded 0.5 cm or when the fruit surface exhibited pronounced cracking, softening, or withering.

(5) $$\mathrm{Decay}\;\mathrm{rate}\;(\%)=\frac{Number\;of\;decay\;tomatoes}{20}\times 100\%$$

### 2.6. The Inhibitory Effect Test of NED on Cherry Tomato Surface Microorganisms

Cherry tomatoes subjected to surface disinfection were inoculated by immersion in a suspension of *S. aureus* for 40 min. After surface moisture had evaporated, the fruits were immersed in either sterile water or NED solutions at varying concentrations (1, 2, and 4 μL/mL) for 1 h, then transferred into sterile sampling bags and stored at 25 °C for 1–5 d. The microorganisms adhering to the fruit surfaces were recovered by repeated rinsing with sterile peptone solution, and the pooled washing fluids were combined to yield 40 mL of bacterial suspension, which was then subjected to 10^n^-fold dilution (where n denotes the number of storage days) and plated onto LB agar plates, followed by incubation at 37 °C for 14 h, after which the colony-forming units were enumerated and the corresponding microbial concentration was calculated according to Equation (3).

### 2.7. The Effect of NED Treatment on the Nutritional Content of Cherry Tomatoes

Following the storage experiments, cherry tomatoes were dried in an oven at 40 °C and ground into powder for subsequent nutritional analyses. With reference to the method reported by published studies with appropriate modifications [50,51,52], 1 g of powder was subjected to extraction using 5 mL of different solvents for the respective target components. Soluble sugars were extracted by boiling with distilled water for 30 min, soluble proteins were extracted with 0.1 M sodium chloride solution by grinding on ice for 10 min, total phenolics and flavonoids were extracted with 1% hydrochloric acid in methanol assisted by ultrasonication for 30 min, and vitamin C (VC) was extracted with 50 g/L trichloroacetic acid (TCA) solution by grinding on ice for 5 min. All extracts were clarified by centrifugation, and the supernatants were collected for further analysis.

The soluble sugar content was determined by the phenol–sulfuric acid method, using sucrose as the standard for calibration curve construction. Briefly, 100 μL of the extract diluted 1000-fold was mixed sequentially with 50 μL of phenol (0.09 g/mL) and 250 μL of concentrated sulfuric acid. After incubation at 25 °C for 30 min, the absorbance at 485 nm (A_485_) was recorded. The content was calculated according to Equation (6).

(6) $$\mathrm{Soluble}\;\mathrm{sugar}\;\mathrm{content}\;(\%)=\frac{m^{\prime }\times V\times N}{V_{r}\times m\times 10^{3}}\times 100\%$$

where *m*′ represents the sucrose equivalent in the extract, V is the total volume of the extract, N is the dilution fold, V_r_ is the volume of extract used in the reaction system, and *m* is the sample mass.

Soluble protein content was measured using the Coomassie Brilliant Blue method, with bovine serum albumin (BSA) as the standard for calibration. An aliquot of 40 μL of the protein extract diluted 2-fold was reacted with 200 μL of Coomassie Brilliant Blue G-250 solution, and the absorbance at 595 nm (A_595_) was determined. The content was then derived from Equation (7).

(7) $$\mathrm{Soluble}\;\mathrm{protein}\;\mathrm{content}\;(\mathrm{mg}/g)=\frac{m_{B}\times V\times N}{V_{r}\times m\times 10^{3}}$$

where *m_B_* denotes the BSA equivalent obtained from the standard curve, V is the total volume of the extract, N is the dilution fold, V_r_ is the volume of protein solution in the reaction system, and *m* is the sample mass.

For vitamin C quantification, the following reaction system was employed: 100 μL of TCA solution, 100 μL of the VC extract pre-diluted 10-fold, 100 μL of anhydrous ethanol, 50 μL of 4,7-diphenyl-1,10-phenanthroline (5 g/L), and 150 μL of ferric chloride (0.3 g/L) were mixed and incubated at 30 °C for 1 h, after which the absorbance at 534 nm (A_534_) was recorded. A blank was prepared by substituting the VC extract with TCA solution. The VC equivalent was calculated based on the measured A_534_ and a standard curve constructed with ascorbic acid, and the final content was computed using Equation (8).

(8) $$\mathrm{VC}\;\mathrm{content}\;(\mathrm{mg}/100\;g)=\frac{m_{c}\times V\times N}{V_{r}\times m_{s}\times 10^{3}}\times 100$$

where *m_c_* is the VC equivalent derived from the standard curve, V is the volume of the extract, N is the dilution fold, V_r_ is the volume of VC extract in the reaction system, and *m_s_* is the sample mass.

Total flavonoid content was expressed as relative abundance: 200 μL of the extract diluted 20-fold was taken for absorbance measurement at 235 nm (A_235_), and the results were presented as A_235_/g dry matter. Total phenolic content was determined using the Folin–Ciocalteu (FC) assay. An aliquot of 200 μL of the 20-fold diluted extract was mixed with 200 μL of distilled water and 50 μL of FC reagent, allowed to equilibrate at room temperature for 10 min, and then 50 μL of sodium carbonate solution (20%) was added. The mixture was incubated at 35 °C in the dark for 30 min, and the absorbance at 765 nm (A_765_) was measured. Gallic acid (GAE) was employed as the standard for calibration, and the results were expressed as gallic acid equivalents (mg GAE/g dm), calculated according to Equation (9).

(9) $$\mathrm{Total}\;\mathrm{phenolic}\;\mathrm{content}\;(\mathrm{mg}\;\mathrm{GAE}/g\;\mathrm{dm})=\frac{m_{G}\times V\times N}{\mathrm{Vr}\times m\times 1000}$$

where *m_G_* is the gallic acid concentration, V is the volume of extract participating in the colorimetric reaction, N is the dilution factor, V_r_ is the volume of extract in the reaction system, and *m* is the sample mass, while dm denotes dry weight.

The antioxidant capacity of the samples was evaluated by DPPH radical scavenging activity. An aliquot of 150 μL of the total phenolic extract (20-fold diluted) was mixed with an equal volume of DPPH reagent (0.05 mg/mL) and incubated at room temperature in the dark for 30 min, with methanol serving as the solvent blank. The absorbance at 517 nm (A_517_) was measured, and the scavenging rate was calculated according to Equation (10).

(10) $$\mathrm{DPPH}\;\mathrm{radical}\;\mathrm{scavenging}\;\mathrm{activity}\;(\%)=\left(1-\frac{A_{s}-A_{b}}{A_{c}}\right)\times 100\%$$

where A_c_ represents the absorbance of the blank control, *A_s_* denotes the absorbance of the sample, and A_b_ corresponds to the sample background.

### 2.8. Residue Analysis of NED

Residual analysis of NED was carried out by spectrophotometry. On the basis of the full-wavelength scanning results, 245 nm was selected as the detection wavelength, at which a calibration curve correlating NED concentration with absorbance (A_245_) was constructed. Cherry tomatoes that had been treated with NED and subsequently stored for 7 d were gently blown dry with a stream of air to remove surface moisture and then weighed. The fruits were subsequently rinsed with distilled water at a volume of 200 mL per wash, and the resulting rinsates were collected for absorbance measurement at 245 nm, from which the residual amount of NED on the fruit surface was calculated according to Equation (11).

(11) $$\mathrm{Residual}\;\mathrm{amount}\;(\mathrm{mg}/\mathrm{kg})=\frac{V\%\times 200\times 0.01}{m}\times 1000$$

Here, *V*% represents the concentration of NED in the cleaning solution obtained from the standard curve and A_245_ and m represents the sample mass.

### 2.9. Statistical Analysis

Statistical analyses were conducted using SPSS (Chicago, IL, USA, 19.0). Differences among treatment groups were evaluated by one-way analysis of variance (ANOVA) followed by Tukey’s HSD post hoc test for multiple comparisons. *p* < 0.05 was considered statistically significant. Data processing and basic calculations were performed using WPS Office (version 12.1.0), and graphical representations were generated with WPS Office.

## 3. Results and Discussion

### 3.1. Preparation of NED

**Figure 1:**
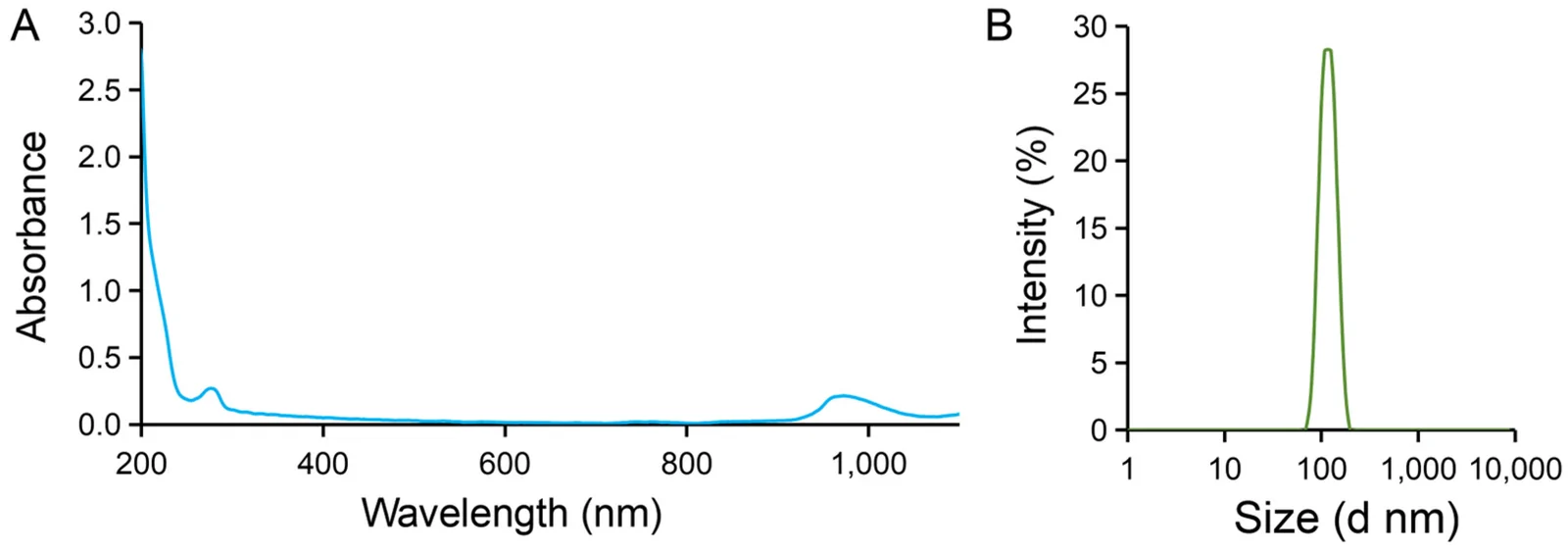
Characterization of NED. (**A**) UV-Vis full-wavelength scanning spectrum. (**B**) Dynamic light scattering (DLS) particle size distribution profile.

The NED was successfully fabricated via high-pressure homogenization, yielding a milky white liquid formulation that exhibited favorable water dispersibility. Full-wavelength scanning revealed a characteristic absorption peak at 245 nm, which could be assigned to the conjugated system and the phenolic hydroxyl group within the 2,4-DTBP molecule. This optical property provided a reliable basis for the qualitative and quantitative analysis of the compound and laid the theoretical foundation for the subsequent establishment of residual detection methods. Particle size and zeta potential measurements indicated that the mean diameter of the nanodroplets fell within the range of 80–170 nm (Figure 1), with a polydispersity index (PDI) of 0.2389, suggesting a relatively uniform size distribution. The zeta potential was determined to be 1.16 mV, and the formulation exhibited good stability, which may be attributable to the steric hindrance effect imparted by the nonionic surfactant Tween 80. Similar phenomena have been documented in other Tween 80-stabilized nanodelivery systems, wherein steric hindrance has been identified as a critical factor in maintaining the physical stability of nanoemulsions [53].

### 3.2. Antimicrobial Activity of NED

**Figure 2:**
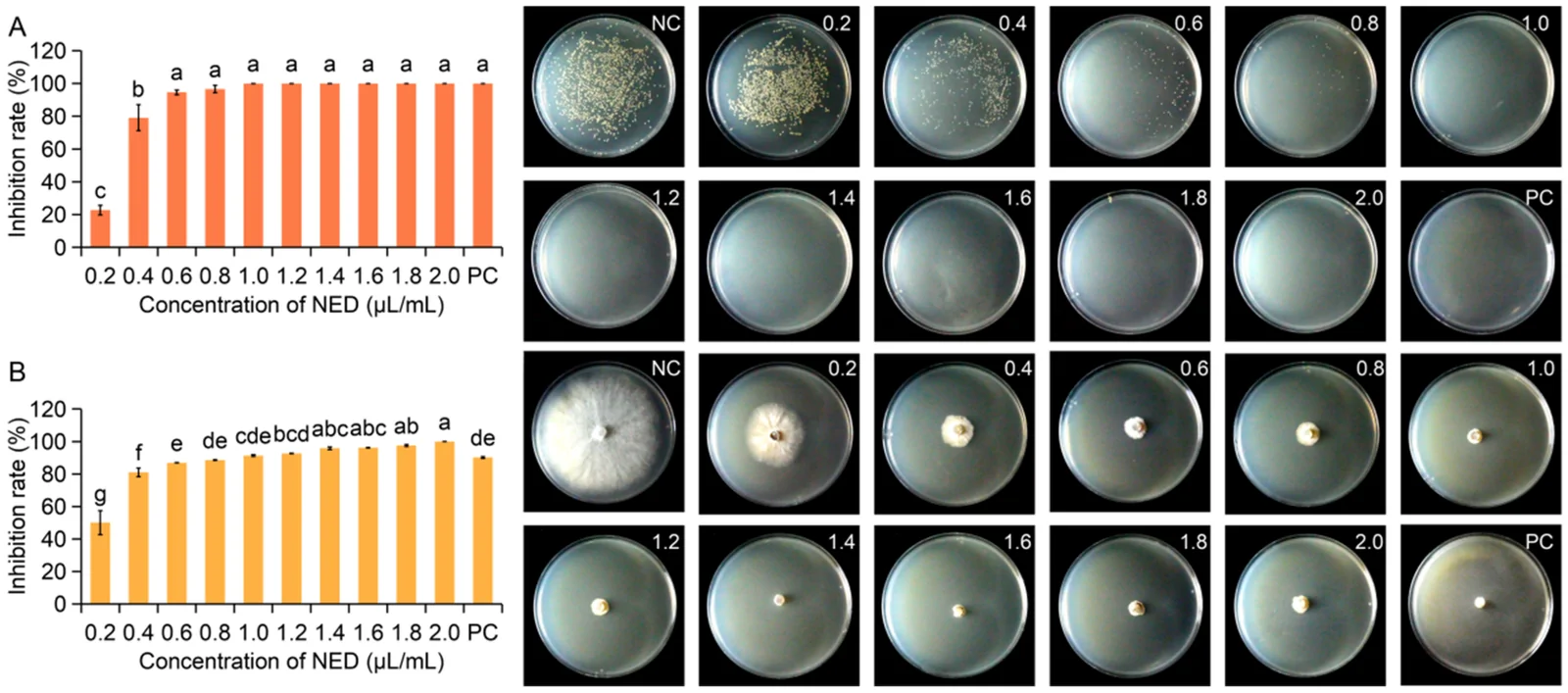
In vitro antimicrobial activity of NED against the pathogenic microorganisms. (**A**) Inhibition rates and corresponding colony morphologies of *S. aureus*. (**B**) Mycelial growth inhibition rates and corresponding colony appearances of *B. cinerea*. NC, negative control; PC, positive control. Different lowercase letters indicate significant differences among treatments (*p* < 0.05).

The NED exhibited potent antimicrobial activity against both *S. aureus* and *B. cinerea*, with the inhibitory efficacy increasing in a concentration-dependent manner. As depicted in Figure 2, at lower concentrations, the antibacterial effect of NED against *S. aureus* was less pronounced than its antifungal activity against *B. cinerea*; however, upon elevation of the concentration, the inhibitory action against the former surpassed that against the latter. Specifically, at 0.2 μL/mL, the inhibition rate against *S. aureus* reached 22.76%, whereas that against *B. cinerea* exceeded 50.04%. In terms of minimum inhibitory concentration (MIC), the values were determined to be 1.0 μL/mL for *S. aureus* and 2.0 μL/mL for *B. cinerea*. This phenomenon suggests that the two pathogenic microorganisms possess distinct concentration thresholds for susceptibility to NED, fungi can be partially inhibited at lower concentrations, yet the concentration required for complete inhibition is higher than that for bacteria. This discrepancy may be associated with the intrinsic differences between Gram-positive bacteria and filamentous fungi in terms of cell wall architecture, membrane composition, and efflux pump systems, although the underlying molecular mechanisms warrant further investigation. At a concentration of 1.0 μL/mL, NED achieved an inhibitory effect against *S. aureus* comparable to that of the positive control, while at concentrations above 1.4 μL/mL, its antifungal activity against *B. cinerea* surpassed that of the positive control. Collectively, these findings substantiate the potential of NED as a potent antimicrobial agent.

### 3.3. Preservation Efficacy of NED

**Figure 3:**
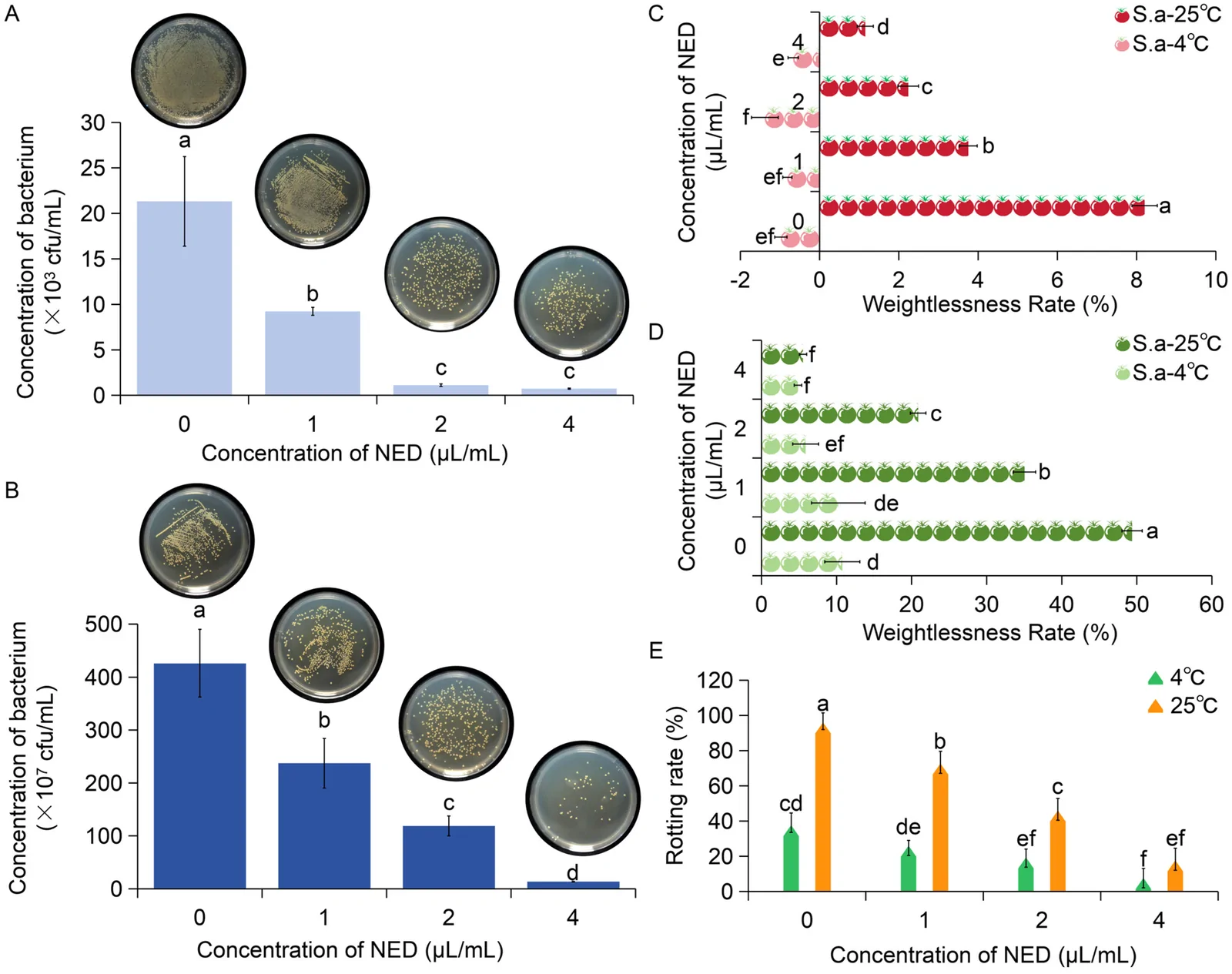
Evaluation of the preservative efficacy of NED on postharvest cherry tomatoes. (**A**) Quantification of surface microbial loads on cherry tomatoes stored at 4 °C for 7 d, with representative plate images shown in the insets. (**B**) Quantification of surface microbial loads on cherry tomatoes stored at 25 °C for 7 d, with representative plate images shown in the insets (bacterial suspensions diluted 10^5^-fold). (**C**) Weightlessness rates of cherry tomatoes inoculated with *S. aureus*. (**D**) Weightlessness rates of cherry tomatoes inoculated with *B. cinerea*. (**E**) Rotting rate of cherry tomatoes inoculated with *B. cinerea*. Different lowercase letters denote significant differences among groups (*p* < 0.05).

In the postharvest storage experiments on cherry tomatoes, NED demonstrated pronounced preservative effects under both low-temperature (4 °C) and ambient (25 °C) conditions, a conclusion jointly substantiated by the three core indicators of microbial load, weight loss, and decay incidence. Figure 3A,B illustrate the microbial loads on fruit surfaces following treatment with varying concentrations of NED and subsequent storage at the two temperatures for 7 d. In the water-treated control groups, the surface microbial concentrations reached approximately 2 × 10^4^ and 4 × 10^9^ CFU at 4 °C and 25 °C, respectively, whereas NED treatment resulted in a substantial reduction, 1 μL/mL sufficed to decrease the microbial population by over 50%, and at 4 μL/mL, the counts fell to 7 × 10^2^ and 1 × 10^8^ CFU at the respective temperatures. Overall, NED exhibited more potent antibacterial efficacy at 25 °C, achieving a 97% reduction relative to the control, compared with a 65% reduction at 4 °C.

Figure 3C,D present the weightlessness rates of the fruits. Notably, after 7 d of storage at 4 °C, the mass of inoculated tomatoes remained virtually unchanged, with no statistically significant difference discernible between the NED-treated and control groups. In contrast, at 25 °C, the control group registered a weight loss of 8.20%, while NED treatment significantly mitigated this loss; most strikingly, the 4 μL/mL concentration resulted in a weight loss of merely 1.15%. In comparison with *S. aureus*-inoculated fruits, those infected with *B. cinerea* exhibited higher weightlessness rate during storage, peaking at 49.36%, which was observed in the 25 °C control group; this parameter declined markedly with increasing NED concentrations, presumably attributable to the fungistatic action of NED that curtails fungal proliferation and consequently reduces organic matter consumption. Remarkably, the preservative effect of 4 μL/mL NED at ambient temperature (weight loss of 5.53%) even surpassed that of low-temperature storage alone (weight loss of 10.76%), suggesting that this nanoemulsion holds promise as a substitute for, or partial supplement to, cold-chain preservation. It is an implication of considerable practical significance for reducing energy consumption during postharvest storage and transportation.

Furthermore, under ambient storage, cherry tomatoes in the water-treated control group infected with *B. cinerea* displayed conspicuous deterioration, including juice exudation, tissue wilting, and rotting, as a consequence of fungal destruction. By contrast, NED-treated fruits manifested markedly milder disease symptoms. As depicted in Figure 3E, the decay incidence of the 25 °C water-treated control reached as high as 96.67%, whereas that of the nanoemulsion-treated group plummeted to 18.33%. Given that low temperature inherently suppresses pathogen growth, the decay rates of tomatoes stored at 4 °C were generally low, falling below 30%. With the additional preservative action of NED, the decay incidence was further reduced to 8.33%, manifesting a synergistic advantage conferred by the combined application of low temperature and antimicrobial agents. Collectively, these lines of evidence corroborate the considerable potential of the as-prepared nanoemulsion for application in postharvest preservation of fruits.

### 3.4. Inhibitory Effect of NED on Cherry Tomato Surface Microorganisms

**Figure 4:**
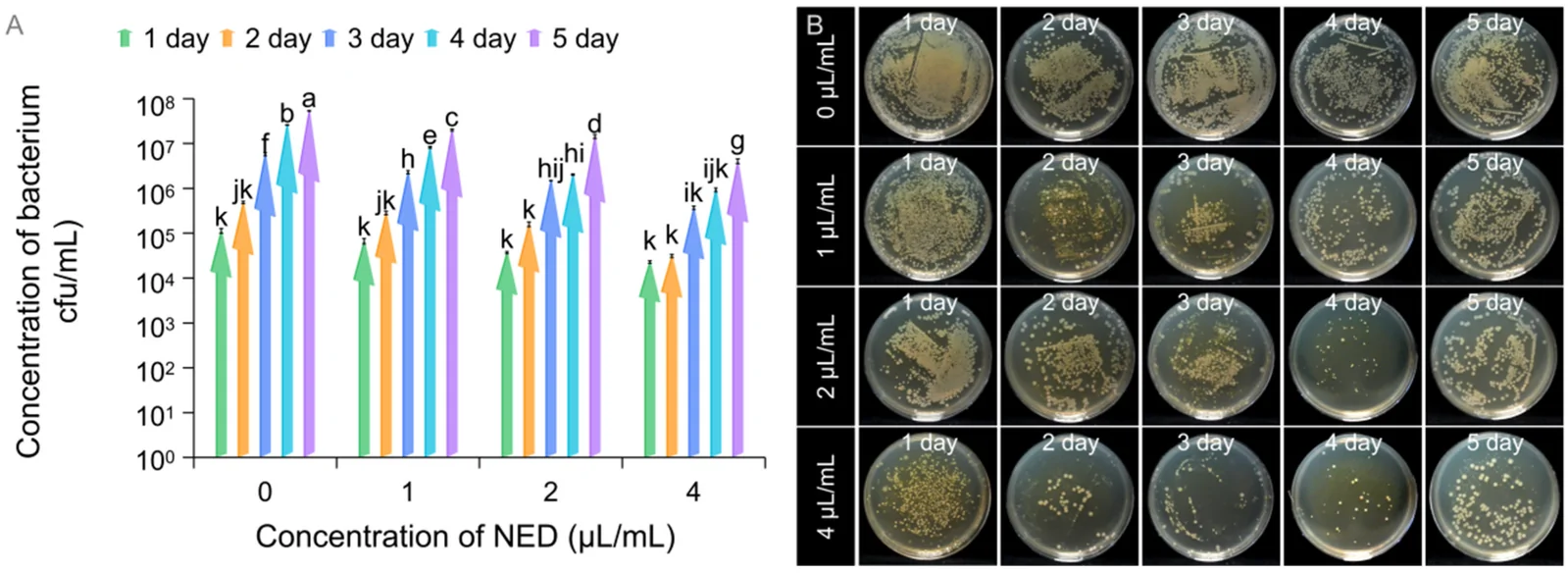
Dynamic influence of NED treatment on surface microbial proliferation on cherry tomatoes during storage. (**A**) Temporal changes in surface microbial loads on cherry tomatoes for 1 to 5 d. Different lowercase letters denote significant differences (*p* < 0.05). (**B**) Representative photographs of plate spreading (aliquots sampled on day n were diluted 10^n^-fold prior to plating).

On the basis of the preservative efficacy exhibited by the 2,4-DTBP nanoemulsion, we further investigated its influence on the microbial population dynamics on fruit surfaces, with the results summarized in Figure 4. Irrespective of whether the fruits belonged to the control or treatment groups, the surface microbial loads increased progressively with prolonged storage time. Analysis along the concentration axis revealed that fruits pre-treated with nanoemulsions had significantly fewer surface microorganisms than their control counterparts, and this reduction exhibited a clear concentration-dependent pattern; higher application concentrations were associated with lower microbial counts. During the later stages of storage (days 4 to 5), the difference in bacterial concentration between the treatment and control groups reached up to two orders of magnitude, and even at the earlier time points (days 1 to 3), a disparity of at least one order of magnitude was consistently observed. These findings further corroborate the potent antimicrobial activity of NED and underscore its promising potential as a postharvest preservative for food products.

### 3.5. Nutrient Preservation Effects of NED on Cherry Tomatoes

**Figure 5:**
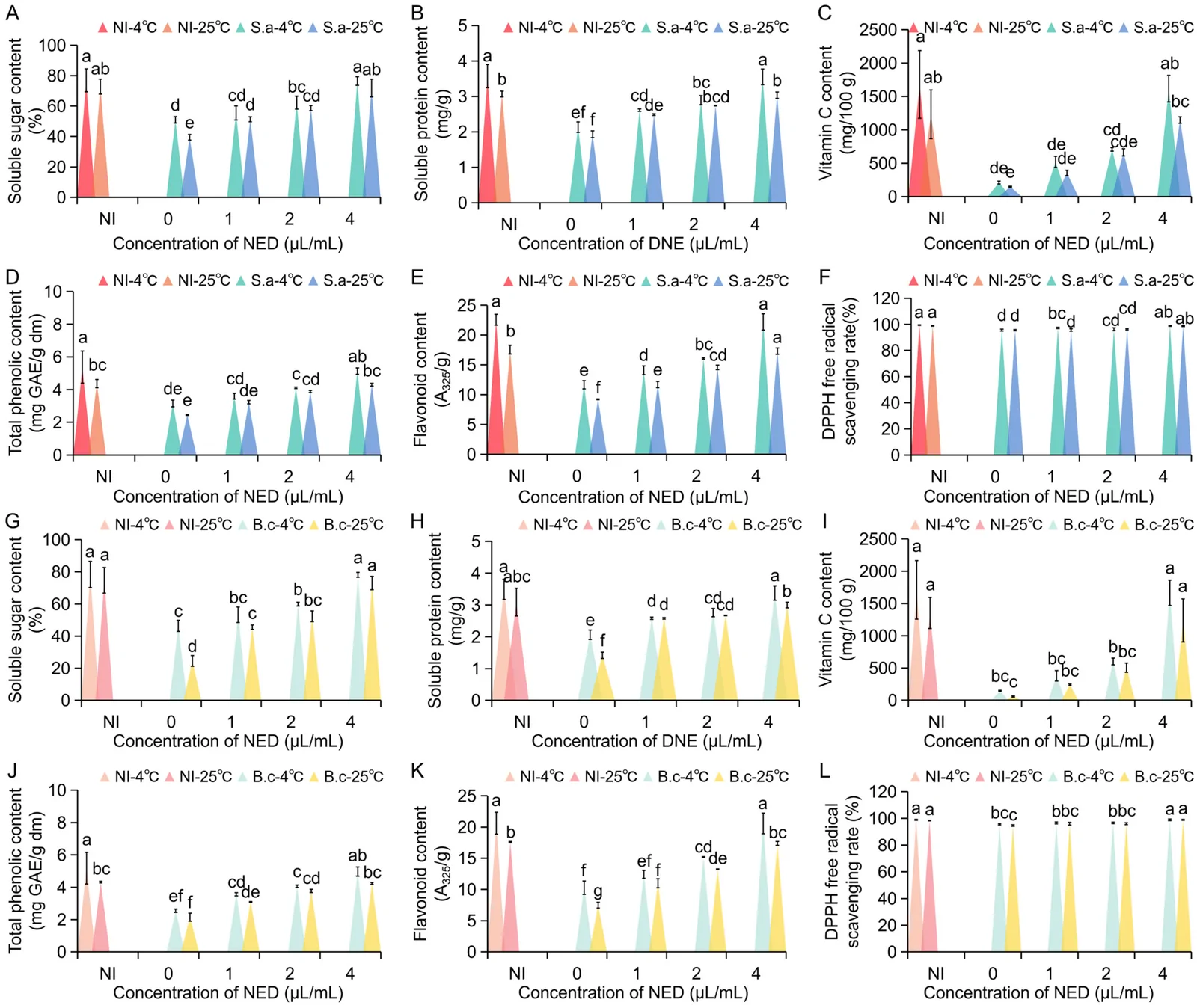
Protective effects of NED on nutritional quality and antioxidant activity. (**A**–**F**) Determinations of various quality parameters in fruits inoculated with *S. aureus* after 7 d of storage: soluble sugars (**A**), soluble proteins (**B**), vitamin C (**C**), total phenolics (**D**), total flavonoids (**E**), and DPPH radical scavenging activity (**F**). (**G**–**L**) Determinations of various quality parameters in fruits inoculated with *B. cinerea*: soluble sugars (**G**), soluble proteins (**H**), vitamin C (**I**), total phenolics (**J**), total flavonoids (**K**), and DPPH radical scavenging activity (**L**). NI represents no inoculation. Different lowercase letters indicate significant differences among treatments (*p* < 0.05).

The efficacy of NED in preserving the nutritional quality of cherry tomatoes was systematically evaluated using soluble sugars, soluble proteins, vitamin C, total phenolics, and flavonoids as assessment indices, complemented by DPPH radical scavenging activity as an indicator of antioxidant capacity. As shown in Figure 5A,G, the soluble sugar content of the non-inoculated blank control group (NI) was significantly higher than that of all pathogen-challenged groups, with peak values observed under 4 °C storage (76.91% and 78.30%, respectively). *S. aureus* inoculation provoked a substantial decline in soluble sugar content (50.96% at 4 °C and 39.47% at 25 °C), whereas *B. cinerea* challenge caused even more pronounced losses (46.39% at 4 °C and 24.51% at 25 °C), both being significantly inferior to the non-inoculated group. Nevertheless, irrespective of the pathogen species or storage temperature, the NED-treated groups exhibited higher soluble sugar contents than their respective controls. Furthermore, as the treatment concentration was elevated from 1 to 4 μL/mL, the soluble sugar content in each challenged group increased in a concentration-dependent fashion, suggesting that NED effectively curtailed the sugar depletion triggered by pathogen infection. Notably, the combined application of NED and low temperature enabled the soluble sugar content of cherry tomatoes to be maintained at a level comparable to that of microbial contamination-free samples.

The variation pattern of soluble proteins closely mirrored that of soluble sugars (Figure 5B,H). Under 4 °C storage, the NI control group registered the highest soluble protein contents (3.57 and 3.49 mg/g). For the water-treated control groups challenged with *S. aureus* and *B. cinerea*, the protein contents descended to their lowest levels, reaching 2.13 and 1.93 mg/g at 4 °C and 25 °C for the former, and 2.07 and 1.42 mg/g for the latter. However, NED application markedly reversed protein degradation, with protein contents exhibiting a sustained upward trend in response to increasing NED concentrations. The 4 μL/mL NED-treated groups attained the highest values among all challenged groups (3.55 and 3.03 mg/g for *S. aureus*-challenged fruits at the two temperatures, and 3.38 and 3.00 mg/g for *B. cinerea*-challenged ones), which were not statistically distinguishable from those of the non-inoculated groups. These findings indicate that NED alleviates the decomposing effects of pathogens on sample proteins, thereby preserving protein stability during storage.

Vitamin C, a core indicator for evaluating postharvest quality of fruits and vegetables, exhibited concentration changes as depicted in Figure 5C,I. The analytical results revealed that both bacterial and fungal infections induced substantial oxidative losses of VC, with fungal challenge inflicting greater damage and more pronounced depletion. The *B. cinerea*-challenged group without NED supplementation recorded the lowest VC contents (145.30 and 57.52 mg/100 g), followed by the *S. aureus*-challenged group (210.00 and 147.77 mg/100 g), representing a reduction exceeding 80% relative to the non-inoculated group. This phenomenon may be attributed to the competitive nutrient consumption by fungal growth, coupled with the release of reactive oxygen species that accelerate VC oxidation. Nevertheless, exogenous application of NED significantly retarded vitamin C oxidative degradation, and the protective effect strengthened with increasing NED concentration, the 1 and 2 μL/mL NED treatment groups exhibited gradual recoveries of vitamin C levels, while the 4 μL/mL group approached the levels of the non-inoculated blank group.

Total phenolics and total flavonoids, as core functional components of cherry tomatoes, are presented in Figure 5D,E,J,K for the various treatment groups. Specifically, the non-inoculated tomatoes stored at 4 °C exhibited the highest contents of both total phenolics (5.37 and 5.18 mg GAE/g dm) and total flavonoids (22.57 and 20.61 A_325_/g) among all groups. Microbial infection triggered substantial degradation of phenolic and flavonoid compounds, at 25 °C; both indices in the 0 μL/mL NED groups decreased by more than 50%, with *S. aureus*-infected tomatoes dropping to 2.46 mg GAE/g dm and 9.24 A_325_/g, and *B. cinerea*-infected ones further declining to their lowest values of 2.15 mg GAE/g dm and 7.47 A_325_/g. Under 4 °C storage, the protective effect of low temperature attenuated the extent of wastage relative to ambient conditions, though the declines still exceeded 40%. Following graded NED treatment, both total phenolic and total flavonoid contents rose significantly with increasing NED concentrations, the 4 °C-stored tomatoes treated with 4 μL/mL NED attained peak values within the challenged groups for total phenolics (5.13 and 4.97 mg GAE/g dm) and total flavonoids (22.22 and 20.56 A_325_/g), showing no significant divergence from the non-inoculated group. These findings confirm that pathogen infection accelerates the consumption of endogenous phenolic and flavonoid compounds, whereas NED effectively mitigates their degradation, thereby preserving the pool of antioxidant-active substances in the samples.

Subsequent antioxidant activity assays corroborated similar conclusions (Figure 5F,L). No significant difference in radical scavenging activity was observed between the non-inoculated blank groups stored at 4 °C and 25 °C (scavenging rates exceeding 98%), indicating that temperature exerts a limited influence on basal antioxidant capacity in the absence of pathogen stress. The blank groups (0 μL/mL NED) challenged with *S. aureus* and *B. cinerea* exhibited markedly reduced scavenging rates of approximately 95%, reflecting the attenuating effect of pathogen stress on the antioxidant potential of the samples. Upon NED application, DPPH scavenging activity recovered in a dose-dependent manner, with the 4 μL/mL NED-treated group approaching the levels of the blank control. Taken together with the total phenolic and flavonoid results, it can be inferred that NED alleviates the pathogen-induced decline in antioxidant capacity by sustaining the endogenous levels of phenolic and flavonoid antioxidants.

Postharvest pathogen infection not only compromises the visual quality of fruits but also induces severe nutritional deterioration. The present study revealed that challenge with either *S. aureus* or *B. cinerea* led to substantial reductions in the contents of soluble sugars, soluble proteins, vitamin C, total phenolics, and total flavonoids in cherry tomatoes, with fungal infection inflicting more severe damage than bacterial infection. This disparity may be ascribed to the invasive growth mode of fungi, whose hyphae penetrate cell walls and secrete extracellular enzymes and mycotoxins, resulting in more thorough destruction of cellular architecture, while concurrently releasing reactive oxygen species that accelerate the oxidative depletion of antioxidant compounds [54]. Upon NED application, all aforementioned nutritional parameters recovered in a concentration-dependent manner, with the 4 μL/mL treatment groups exhibiting no statistically significant differences from the non-inoculated blank groups for most indices. Considering the antimicrobial action of NED, it can be inferred that the nutrient-preserving effect of NED does not arise from direct modulation of fruit metabolic activities, but rather from the indirect suppression of pathogen proliferation and metabolic activity. This suppression mitigates the consumption of host nutrients and the destruction of cellular structures by pathogens, thereby safeguarding the integrity of fruit tissues and the nutrients contained therein.

### 3.6. Results of Residue Analysis of NED

**Figure 6:**
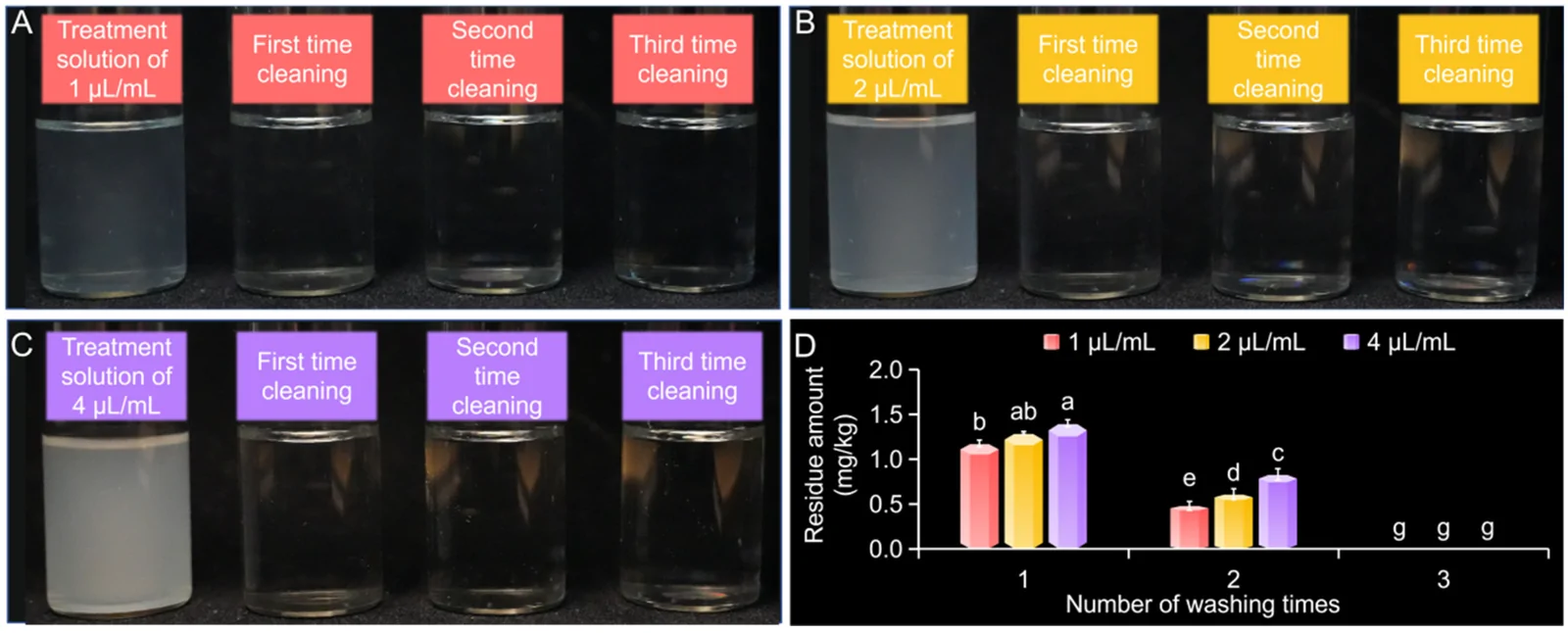
Visual observation of cleaning solutions and quantitative detection of NED residue on cherry tomato fruit. (**A**) Treatment group with 1 μL/mL NED. (**B**) Treatment group with 2 μL/mL NED. (**C**) Treatment group with 4 μL/mL NED. (**D**) Quantification of NED residue (mg/kg) on fruit surface. Different lowercase letters indicate significant differences among groups (*p* < 0.05).

Using 245 nm as the detection wavelength, the residual amounts of NED on the surface of cherry tomatoes treated with varying concentrations of the nanoemulsion and subsequently stored for 7 d were quantitatively analyzed by means of the NED standard curve. The visual appearance of the washing solutions and the corresponding residual data are compiled in Figure 6. In terms of the appearance of the solutions, the NED treatment solutions exhibited varying degrees of milky whiteness, with the color intensity deepening progressively as the applied concentration increased. In contrast, the rinsates collected after water washing from all treatment groups appeared clear and transparent. The quantitative residual detection results (Figure 6D) revealed that, under identical washing frequencies, a significant positive correlation existed between the residual NED load recovered from fruit surfaces and the applied concentration. After a single rinse, the residual amounts for the 1, 2, and 4 μL/mL treatment groups reached 1.17, 1.29, and 1.40 mg/kg, respectively. Following a second water wash, the residual NED levels recovered from fruit surfaces in all three groups decreased markedly to 0.48, 0.61, and 0.83 mg/kg, corresponding to removal rates of 58.97%, 52.71%, and 40.71% relative to single rinsing, which indicated that samples treated with lower NED concentrations exhibited higher residual removal efficiency. Upon completion of the third water rinse, the residual NED amounts recovered from fruit surfaces in all three treatment groups fell below the detection limit of the spectrophotometric method, demonstrating that three washes with water sufficed to reduce surface NED residues to a level no longer detectable by the current analytical approach. Taken together, the visual images of the washing solutions and the quantitative data provide evidence that water rinsing effectively removes surface-adherent NED from fruits, and the efficacy of residual elimination is significantly enhanced with increasing washing frequency. Under identical washing conditions, higher NED application concentrations resulted in greater residual loads recovered after a single wash, yet three repeated washes were sufficient to reduce the recoverable epidermal residues from all concentration treatments to below the detection limit.

From a physicochemical perspective, the likelihood of NED penetration into the fruit interior is expected to be low. Previous studies on plant cuticle ultrastructure have revealed that the tomato fruit cuticle possesses interplanar spaces of approximately 0.45–10 nm [55], and the maximum pore radius of plant cuticles has been generally estimated to range from 0.3 to 2.12 nm across different species [56]. These dimensions are substantially smaller than the average droplet size of NED (80–170 nm), suggesting that intact nanoemulsion droplets are unlikely to traverse the cuticular barrier. Additionally, 2,4-DTBP is highly hydrophobic, which favors its partitioning into the lipophilic wax layer of the cuticle rather than its migration into the hydrophilic pulp tissue via an aqueous medium. Nevertheless, it should be emphasized that these arguments are based on theoretical inference from the physicochemical properties of NED and the structural characteristics of the tomato cuticle, rather than on direct experimental evidence. It is therefore acknowledged that the present analysis was performed on the washing solutions rather than on the fruit tissues themselves. Future work employing more sensitive analytical techniques on whole-fruit homogenates is warranted to definitively confirm the absence of internal residues.

### 3.7. Limitations of the Study

Several limitations of this study should be acknowledged. First, the proposed antimicrobial mechanism of NED was primarily inferred from in vitro antimicrobial assays, and direct in situ ultrastructural evidence is still lacking. Second, the preservative efficacy was evaluated only under controlled laboratory conditions, and further validation under commercial packinghouse or field conditions is necessary to confirm the practical applicability of NED. In addition, while the washing experiments demonstrated that surface NED could be removed by rinsing, the present study did not directly determine the amount of 2,4-DTBP that might have migrated into the fruit pulp. The safety conclusion drawn in this work is therefore based on indirect evidence of removability and should be further substantiated through whole-fruit residue analysis using more sensitive analytical techniques in future investigations. Finally, the potential impact of NED treatment on the sensory attributes of cherry tomatoes, which are critical for consumer acceptance, has not yet been assessed and warrants future investigation.

## 4. Conclusions

In summary, this study successfully established a laboratory-scale nanoemulsion delivery system loaded with 2,4-di-tert-butylphenol (NED), which effectively addressed the water solubility limitation of this natural phenolic compound under controlled experimental conditions. The formulation exhibited a uniform particle size distribution, favorable water dispersibility, and reliable physical stability. In vitro antimicrobial assays confirmed that NED exerts concentration-dependent inhibitory effects against the primary postharvest spoilage organisms of cherry tomatoes, with the MIC value against *S. aureus* (1.0 μL/mL) being lower than that against *B. cinerea* (2.0 μL/mL), and its efficacy at higher concentrations even exceeding that of the respective positive control drugs under the specific conditions tested.

Postharvest preservation experiments demonstrated that NED effectively reduced surface microbial loads, suppressed weight loss, and markedly decreased decay incidence under both low-temperature and ambient storage conditions in laboratory settings. Its protective effects on nutritional components, including soluble sugars, soluble proteins, vitamin C, total phenolics, and total flavonoids were likely achieved indirectly through the inhibition of pathogen metabolic activities. With the concomitant preservation of antioxidant capacity contributing to the maintenance of postharvest quality. Safety evaluation indicated that three washes with water completely removed NED residues from fruit surfaces, leaving no detectable amounts, thereby supporting its potential safety for future applications. Collectively, these findings suggest that NED is a promising candidate as a naturally derived antimicrobial nanoformulation for postharvest preservation of fruits and vegetables. However, as the current results were obtained exclusively under laboratory conditions, further validation through pilot-scale or commercial packinghouse trials is essential before drawing definitive conclusions regarding its efficacy in real-world applications. Nevertheless, this work provides a fundamental basis for the further development and optimization of nanoencapsulated natural phenolic compounds in the field of postharvest preservation.

## Data Availability

The original contributions presented in this study are included in the article. Further inquiries can be directed to the corresponding authors.
